# Plant-Based Bimetallic Silver-Zinc Oxide Nanoparticles: A Comprehensive Perspective of Synthesis, Biomedical Applications, and Future Trends

**DOI:** 10.1155/2022/1215183

**Published:** 2022-04-30

**Authors:** Maria Ehsan, Abdul Waheed, Abd Ullah, Abeer Kazmi, Amir Ali, Naveed Iqbal Raja, Zia-ur-Rehman Mashwani, Tahira Sultana, Nilofar Mustafa, Muhammad Ikram, Huanyong Li

**Affiliations:** ^1^Department of Botany, PMAS-Arid Agriculture University Rawalpindi, Pakistan; ^2^Xinjiang Key Laboratory of Conservation and Utilization of Plant Gene Resources, Xinjiang Institute of Ecology & Geography, Chinese Academy of Sciences, Urumqi 830011, China; ^3^Xinjiang Key Laboratory of Desert Plant Roots Ecology and Vegetation Restoration, Xinjiang Institute of Ecology and Geography, Chinese Academy of Sciences, Urumqi 830011, China; ^4^Cele National Station of Observation and Research for Desert-Grassland Ecosystems, Cele 848300, China; ^5^College of Plant Science and Technology, Huazhong Agricultural University, Wuhan 430070, China; ^6^Department of Biotechnology, Faculty of Chemical and Life Sciences, Abdul Wali Khan University Mardan (AWKUM), Mardan, Pakistan; ^7^Department of Genetics, Institute of Hydrobiology, University of Chinese Academy of Sciences (UCAS), Wuhan, China; ^8^Binzhou Vocational College, Binzhou 256603, China

## Abstract

The universal emphasis on the study of green nanotechnology has led to biologically harmless uses of wide-ranged nanomaterials. Nanotechnology deals with the production of nanosized particles with regular morphology and properties. Various researches have been directed on nanomaterial synthesis by physical, chemical, and biological means. Understanding the safety of both environment and in vivo, a biogenic approach particularly plant-derived synthesis is the best strategy. Silver-zinc oxide nanoparticles are most effective. Moreover, these engineered nanomaterials via morphological modifications attain improved performance in antimicrobial, biomedical, environmental, and therapeutic applications. This article evaluates manufacturing strategies for silver-zinc oxide nanoparticles via plant-derived means along with highlighting their broad range of uses in bionanotechnology.

## 1. Introduction

Biological approaches engage all those principles and procedures, which are eco-friendly during the assembly of chemicals. The biological method for synthesizing starting chemicals for industrial and agricultural applications has been acknowledged worldwide for its nontoxic effects on the environment. The latest learning of exploration work on nanobiotechnology has guaranteed safe uses of a range of nanoparticles. Nanotechnology generally focuses on the production of different but regular-sized, shaped nanoparticles intending to make them more useful for the betterment of mankind. While on the other hand, physical and chemical approaches have difficulties of being expensive and environmentally hazardous along with producing nanoparticles with distinctive properties and precise morphology. This shows that in nanotechnology biological procedures to synthesize nanomaterials are biologically and environmentally safe applications [1]. After over years of work, nanobiotechnology gets straight to the point where scientists have researched to develop nanomaterials from biomolecules such as proteins, lipids, secondary metabolites, and metals from alive organisms especially plants [2]. Among these biomolecules, metals have been methodically discovered for the production of green nanomaterials. In the application of nanoparticles, bimetallic materials attained more attention because of their synthesis by heterogeneous catalyst [3]. Silver (Ag) and zinc oxide (ZnO) as bimetallic Ag/ZnO nanoparticles are of more importance, owing to industrial, medical, and environmental uses. The work done on the synthesis and application of various metals with zinc oxide nanomaterials has been reviewed through different studies [4–10].

Bimetallic nanoparticles (BNPs) are preferred over monometallic NPs (MNPs) because of their immensely improved optical, catalytic, and biological properties [11]. Two different metal atoms are linked together in a single NP to form BNPs. Apart from the aggregation of traits associated with the presence of two separate metals, it is also expected that BNPs would display unique properties as a result of the synergistic effect of metals [12–14]. The metal atoms' configuration determines the potential characteristics of BNPs. The redox potentials of the metal ions, as well as the type of the reducing agents utilized, strongly impact the orientation of BNPs, which can be core-shell, heterostructure, multishell, cluster-in-cluster, and random alloy [15]. Specifically, the reducing ability of biomolecules is coupled with metals for the production of other economically and environmentally beneficial mono- and/or bimetallic nanomaterials. Among bimetallic nanomaterials, Ag/ZnO nanoparticles have acknowledged much consideration because of their vast range of applications [16]. These engineered nanomaterials Ag/ZnO have dual functional roles, the capability of high-value uses such as analytical and active therapeutic agents for the cure of numerous diseases, photodynamic therapy [17], and photocatalyst [18]. A new exciting property of Ag/ZnO alloy NPs is their imaginative applications due to interaction with light and the creation of vibrant colors. Concerning other nanoparticles, Ag/ZnO alloy nanoparticles could be synthesized by physical, chemical, and biological procedures. By manipulating surface chemistry and morphology during amalgam, electrical to optical properties of Ag/ZnO NPs can be managed. Among biological approaches, researchers showed more interest in plants for the synthesis of nanoparticles. This review article emphasizes the fabrication of clear images of approaches monitored for synthesis and characterization of Ag/ZnO NPs, their wide range of importance, and applications. Moreover, the article discovers diverse paths acquired by biosynthesized Ag/ZnO nanoparticles aimed at showing deadliness during application. It also points out prospects of green synthesized Ag/ZnO nanoparticles concerning its vast significance.

## 2. The Influence of Plant-Derived Ag/ZnO Alloy NPs

Common approaches for synthesizing alloy nanoparticles involved the use of chemical reagents for the reduction of metallic salts into silver and zinc metals. Reducing agents directly affects the size of alloy nanoparticles. The method of synthesizing Ag/ZnO NPs by chemicals led to the creation of less stable nanomaterials, and the outflow of chemical reagents to the surroundings causes harmful effects on the environment and human health. Hence, it is vital to discover substitutive routes for reduction of silver, zinc, and stabilization of their alloy nanoparticles, having the capability of avoiding linked hardships [19]. Recently, microwave solvothermal synthesis approach was also applied to develop Ag/ZnO NPs by using microwave reactor, while zinc acetate dehydrate and silver acetate anhydrous were the reagents [20]. Plant-based compounds likewise their secondary metabolites can be utilized as competent agents for reduction and stabilization, conferring steady size and stable accumulation to synthesized NPs. Sustainable chemistry serves those procedures and principles which are eco-friendly, concerning minimizing leakage of lethal materials commonly used for producing nanomaterials. The biological approach of synthesizing alloy NPs for its vast use has been renowned worldwide due to its harmless effects [21]. Experiments on plant-derived approaches of bimetallic nanoparticles have helped describe novel passages for the production of ranging sizes and morphological shapes of NPs such as cube, rhombic dodecahedron, tetrahedron, octahedron, and spherical, irregular, and crystal-shaped nanomaterials [22]. This could be accomplished by using different plant-based extracts to precursor salts for the production of a diversity of materials having a vast range of applications. Through plant extract, the synthesis of Ag/ZnO NPs is almost the same as utilizing plants to yield Ag/ZnO from silver and zinc oxide salts [23, 24]. In the area of phytomining, direct use of the plant for the production of Ag/ZnO has been advantageous. Plants absorb silver and zinc oxide salts, reduce them, and store them as Ag/ZnO NPs, which could be taken back for further use in eco-friendly means. Concurrently, Ag/ZnO alloy NPs are nontoxic for plants as they do not affect plant growth [25]. Moreover, plant-derived Ag/ZnO NPs most probably stay as lesser aggregate in a mixture form than massive aggregation that occurs due to reagents utilized during chemical approaches of synthesizing nanoparticles. Even though the diversity of sizes still occurs ranging from 1 to 100 nm in Ag/ZnO alloy NPs, small-sized Ag/ZnO NPs are ideal for medical, agricultural, and industrial purposes [26]. Biosynthesized Ag/ZnO NPs from coffee and *Prosopis farcta* used as antimicrobial in the wound healing-medical field. Generally, Ag/ZnO NPs synthesized from greener chemistry assure regularity in size and shape with lesser masses of nanoparticles, along with eco-friendly and improved biodegradable products [27].

## 3. The Followed Means of Plant-Derived Ag/ZnO Alloy NP Synthesis

Besides, the classification of methods was used for preparing NPs into Top-down synthesis and Bottom-up synthesis. However, methods of synthesizing alloy nanoparticles can also be classified into physical, chemical, and biological approaches [27, 28]. Different researches demonstrated Ag/ZnO NP synthesis physically and chemically such as sonochemical precipitation [29–31], radiolytic preparation [32, 33], coprecipitation method [34], low-temperature synthesis [35], solvothermal synthesis [36, 37], polymer-network gel process [38], and combustion synthesis [39]. These methodologies have been progressively more unlikely in terms of safety and noxiousness matters.

The most often exploited chemical and physical approach for the synthesis of BNPs is the reduction of compounds in water-based solutions; however, these synthesis procedures are labour-intensive and expensive and frequently need hazardous/toxic substances. In this aspect, a procedure that is safer, greener, and more environmentally friendly is critical [40, 41]. Bacteria, fungus, yeast, and plants are biological categories that have showed tremendous potential as bioreactors in the synthesis of NPs.

**Figure 1:**
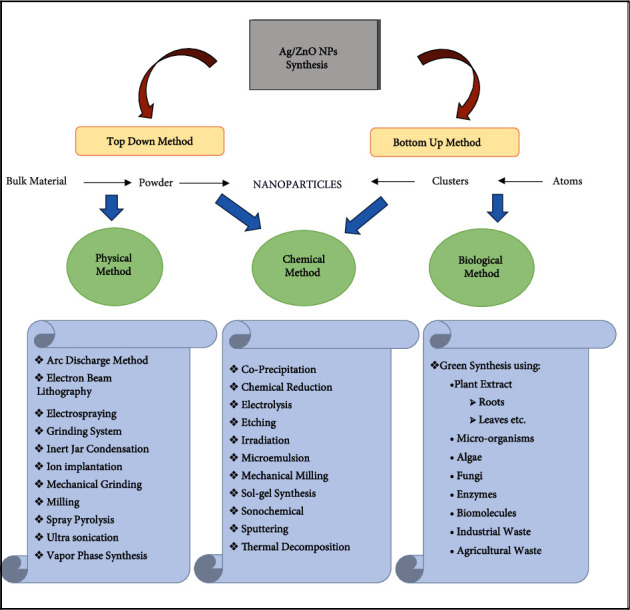
Various routes of Ag/ZnO NP synthesis.

**Figure 2:**
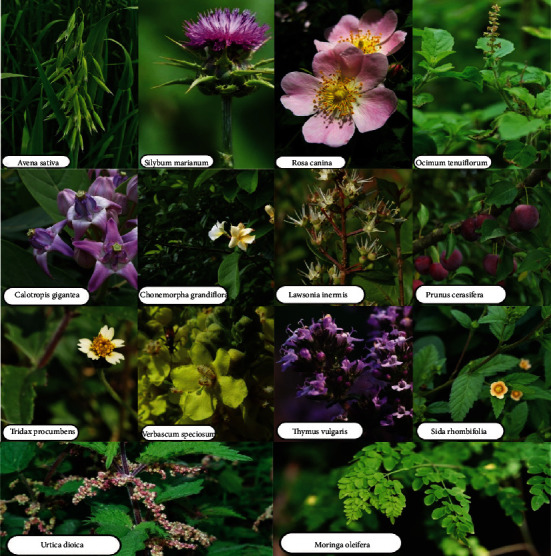
Various plants used in the synthesis of Ag/ZnO nanoparticles.

**Figure 3:**
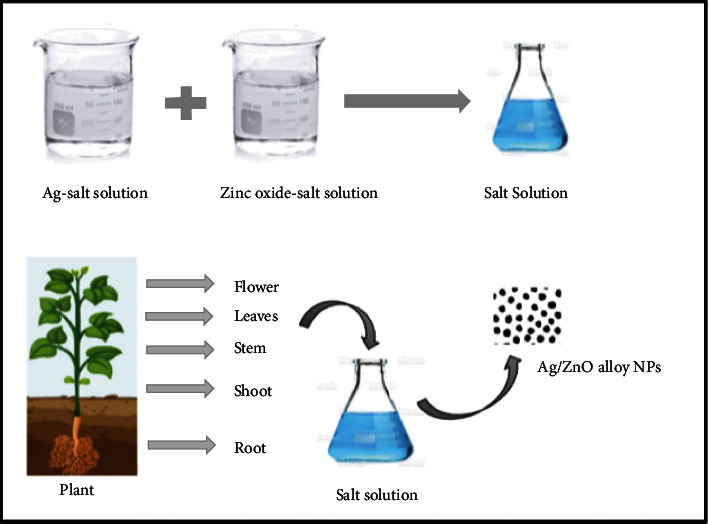
Schematic illustration of different plant parts for the plant-derived synthesis of Ag/ZnO alloy NPs.

Figure 1 shows a flow diagram regarding different means of NP synthesis. The biological method comprises the usage of microbes (bacteria, fungi), algae *Padina gymnospora* [42], and plants for reduction of precursor salts for production of morphologically different nanoparticles. Plants have been shown to be substantial and tremendously advantageous over the others because of their vast availability, low cost, and, most importantly, their safety to living beings and the environment. Plant extracts include secondary metabolites (phytochemicals/bioactive substances) that can decrease metal ions and stimulate the synthesis of NPs with significant and diverse biological and physical activities [43]. Among greener methods of synthesis, plant-based synthesis produces uniform-sized Ag/ZnO NPs.  Figure 2 shows various plants used in the synthesis of Ag/ZnO nanoparticles [44–52]. Moreover, Ag/ZnO NPs could be easily obtained by mixing precursor salts in a beaker with plant extract as a reducing and stabilizing agent, making a sustainable and eco-friendly substitute for metal-based nanoparticle generation, and NPS tend to become more active as a catalytic agent. With the rising confirmation of the significance of plant for synthesizing Ag/ZnO alloy NPs, researches have been conducted with extract of parts like roots, rhizome, shoots, stem, bark, leaves, flowers, and seeds of various plants (Figure 3), utilized for biomolecules like steroids, flavonoids, saponins, alkaloids, and secondary metabolites, having capacity of precursor salt reduction for the production of silver-ZnO nanoparticles [53]. From the beginning of the 21^st^ century, the synthesis of silver-zinc oxide alloy NPs from plants has taken the charge and there are several plants utilized for the generation of silver-zinc oxide nanoparticles ever since [54]. Substances extracted from plants appeared as a very appropriate influencer for the extensive synthesis of alloy NPs else than various described procedures of using risky chemical compounds, causing severe damage if preserve in the environment. The plant material used also affects the consistency and flexibility of NPs [55]. More specifically, the use of peeled *Solanum tuberosum* common tuberous crop was suggested, because of the starch component; a major form of carbohydrate content could be proficiently utilized as a means for NP synthesis [56].

The phytochemicals present in extract like secondary metabolites (phenols, flavonoids, alkaloids, steroids, saponins, etc.) act as reducing as well as capping agents to precursor solution which leads to formation of nanoparticle of different sizes and shapes. Morphology studies show that phytochemicals are effectively controlling the size, shape, and distribution of particles [57].

The morphology of the nanoparticles is also affected by the physical factors such as pH and temperature. The effects of pH and temperature on the biosynthesis of Ag/ZnO NPs were investigated in a recent study to investigate the optimum conditions for producing Ag/ZnO NPs. The results revealed that a lower pH (pH 6 and *T* = 150°C) had a significant impact on the size of the NCs. It appears that at lower pH values, the rate of nucleation is considerably higher, resulting in a large number of ZnO nuclei and, as a result, delayed expansion of the ZnO lattices. The rate of nucleation is low at higher pHs (pH 12 and *T* = 150°C); therefore, the ZnO lattices develop quickly. The precipitation of ZnO nuclei is predicted to begin when the concentration of these Zn^2+^ and OH ions surpasses the critical threshold. This is what is known as the first nucleation step for ZnO production. The rise in thermal energy of the system can be linked to variations in Zn^2+^ and OH ions. It can be deduced from this that the reaction temperature during the synthesis of Ag/ZnO nanostructures has a significant impact in the size modification of the NPs [58]. Temperature, on the other hand, has an impact on the form and size of nanomaterials. At 90, 120, and 150°C, Ag/ZnO NPs were produced. According to the results, increasing the reaction temperature causes smaller Ag/ZnO assemblies to develop. As a result, raising the temperature may cause the Ag/ZnO assemblies to dissociate faster. Zn(OH)_2_ causes the release of free zinc ions Zn^2+^ and hydroxide ions in a regulated manner [59].

**Figure 4:**
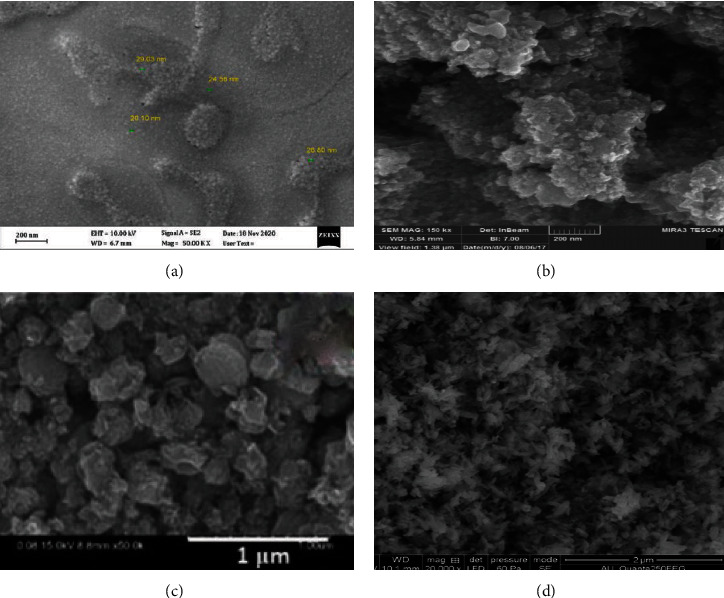
SEM images of different Ag-ZnO NPs. (a) Silver-zinc oxide nanocomposites synthesized using *Pistacia atlantica resin* [173]; (b) synthesis of Ag/ZnO nanoparticles by using aqueous extract of oak fruit hull [26]; (c) silver-zinc oxide nanocomposites synthesized using *Beta vulgaris* (beetroot) extract [103]; (d) silver-zinc oxide nanoparticles synthesized by using *Padina gymnospora* seaweed extract [42].

For characterization of bimetallic nanoparticles, various approaches can be exploited; that is, UV-Vis spectrum confirms synthesis and bimetallic nature of nanoparticles, spectra of EDAX show the existence of certain proportions of precursor element, and SEM determines size and shape of bimetallic nanoparticles and ImageJ software has been in use, analyzing SEM image for particle-size distribution [60] (Figure 4).

## 4. Physiochemical Properties of Ag/ZnO Nanoparticles

As explained previously, certain morphological, physiological, and biochemical properties like an increased surface area to volume ratio, mechanically solid, highly reactive chemically, and photosensitive made these nanoparticles appropriate and distinctive candidates for several medical and therapeutic use [61]. The following headers discuss some of their significantly important properties.

### 4.1. Electronic and Photosensitive Properties

The photosensitive and electronic properties of silver-zinc oxide nanoparticles are symbiotic to a larger magnitude. That is, the photosensitive properties of these plant-based bimetallic nanomaterials depend on the size of particles and show a strong UV-visible spectrum compared with the extinction band that exists on the scale of the bulk metal [62]. When the rate of recurrence of the instance photon is constant with collective excitation of the conducting electrons, it results in the formation of the excitation band and is recognized as localized surface plasma resonance. This resonance leads to the wavelength selection preoccupation with tremendously more coefficient resonance [63]. It is well recognized that the peak of a wavelength of this resonance scale directly depends on morphology (size and shape) and gaps between particles of the nanomaterials along with their electric properties and their native environments like solvents and substrate [64]. These bimetallic colloidal nanoparticles are responsible for the rusty colors in glass windows/doors; monometallic silver and zinc oxide nanoparticles are naturally brown and white. In reality, on the outer surface of these nanoparticles (silver, zinc oxide), free electrons are present that are portable over nanomaterials freely [65]. The mean free path for silver and zinc oxide is less than 50 nm, which is greater than the size of nanoparticles. Hence, upon optic interaction, there is no expectation of scattering from the bulk metals; alternatively, they stand fixed into resonance environments, which is accountable for the localized surface plasma resonance in these bimetallic nanoparticles [66].

### 4.2. Magnetic Properties

The magnetic properties of biogenic nanoparticles are of great interest for researchers from a wide-ranging field, including homo- and heterogeneous catalysis, magnetic fluids, MRI data store, bioremediation like water refinement, and biomedicine. The research work illustrates the following: the size (mean 15 nm) is less than the critical value which executes the best performance of nanoparticles [67]. The magnetic properties of bimetallic nanoparticles conquered efficiently at a low scale, resultantly making these materials priceless, and could be used in various solicitations [68]. The irregular distribution of electronics in nanoparticles is responsible for their magnetic property. The means and methods such as the coreduction method by greener approach and the production of silver-zinc oxide nanoparticles also affect this property [69].

### 4.3. Mechanical Property

The distinctive mechanical properties of greener bimetallic nanoparticles let scientists investigate the new use in numerous areas, like surface engineering, nanobased manufacturing, and nanobased fabrication [70]. Various mechanical attributes like harness, friction, adhesion, stress, and strain and electric modules can be measured to get information about the mechanical nature of greener synthesized silver-zinc oxide nanoparticles thoroughly. Along with these attributes, coagulation, lubrication, and surface coating assist mechanical properties of nanoparticles as well [71]. While comparing with monometallic nanoparticles, microparticles, and bulk metals, it is obvious that bimetallic nanoparticles demonstrate different mechanical properties. Furthermore, during lubrication or grease interaction, when the pressure is large considerably, the distinction in the rigidity and toughness between nanosized particles and the interaction of outer surface controls whether the nanoparticles are concaved into the plan surface or distorted [72]. This significant knowledge could reveal the performance of nanoparticles in contact conditions. Productive results in these areas usually require a deep vision into the fundamentals of the mechanical properties of bimetallic nanoparticles, for example, movement law, interfacial adhesion, hardness, elastic modulus, size-dependent characteristics, and friction [73].

### 4.4. Thermal Properties

It is well recognizing statement that plant-based metallic nanoparticles have thermal conductivity more than solutions in hard form. The thermal conductivity of silver and even oxides like zinc oxide is more than that of engine oil and water at room temperature [74]. Consequently, the solutions having dispersed particles are predicted to showcase increased thermal conductivities comparative to dispersed particles of heat transfer solutions [75]. Nanobased solutions are formed by scattering nanosized particles into a solvent like water, oils, or alcohol. Nanobased solutions are predicted to display greater properties comparative to heat transfer solutions and solutions having particles of microscopic size. As the transfer of heat takes place at the outer surface of the material, it is needed to utilize the nanosized materials with a more surface area to volume ratio. The increased surface area increases the steadiness of suspension as well [76]. In recent times, it has been illustrated that nanobased solutions containing silver and zinc oxide reveal advanced thermal conductivity [77].

## 5. Plant-Derived Ag/ZnO NPs: Uses

**Figure 5:**
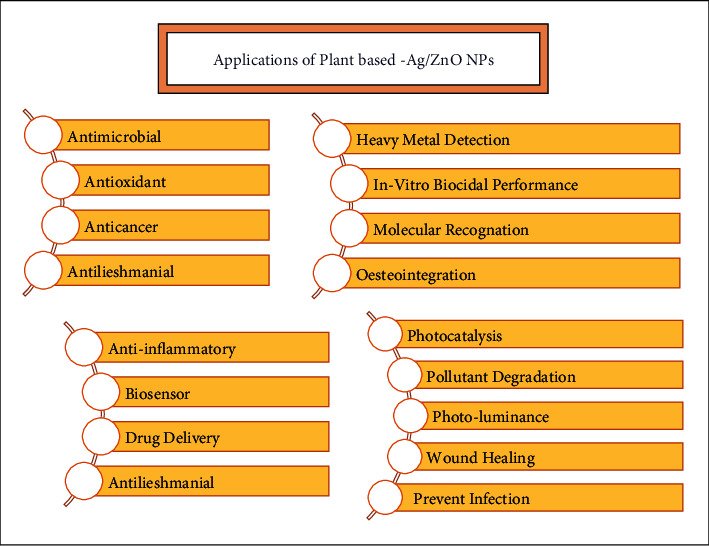
Various applications of plant-based Ag/ZnO NPs described in the literature.

Among alloy nanoparticles, silver-zinc oxide NPs initiate numerous uses including Aesculapian and non-Aesculapian fields.  Figure 5 demonstrates applications of Ag/ZnO alloy nanoparticles. Perhaps, this is due to its distinctive physiological and chemical nature like minimized size, the capability of bonding with biological molecules due to high reactivity, great constancy, and increased surface area-mass ratio, effortless generation and characterization, luminous extension actions, and their reduced toxicity to cells [59, 78–81].

### 5.1. Antimicrobial Use

**Figure 6:**
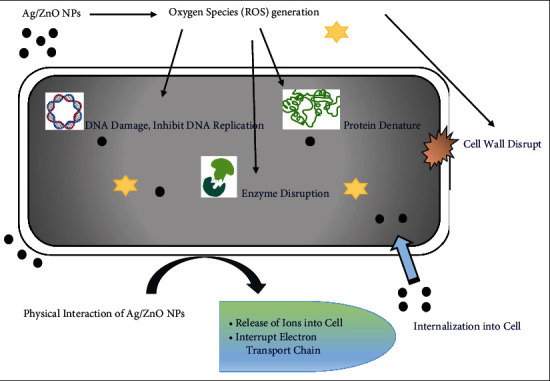
Mechanistic effect of Ag/ZnO NPs on bacterial cell.

Plant-derived Ag/ZnO alloy nanoparticles turned out to be competently stated as an agent that stops microbial growth by killing them. These alloy NPs showed greater antimicrobial activity as compared to monometallic nanoparticles [82]. Ag/ZnO NPs have shown activity against *Micrococcus luteus* and *E. coli* because of their small size and photocatalytic ability [83]. Furthermore, Ag/ZnO NPs also exhibit antibacterial activity against *S. aureus* [39], *P. aeruginosa* [84], *S. epidermis*, *B. subtilis, K. pneumonia*, and *P. aeruginosa*. Therefore, Ag/ZnO NPs came to be hired in studying its effects against microorganisms among different nanomaterials [85]. These NPs established high activity against infective gram-positive and gram-negative bacterial strains.  Figure 6 explains the mechanisms of action of nanoparticles against bacteria [86]. Cell walls and plasma membranes are the main protective hurdle to bacterial tolerance in the surrounding environment. While the absence of peptidoglycan layer in gram –ve bacterial cell wall directed to enhanced bacterial activity on them [87]. There is more generation of reactive oxygen species by NPs because of their higher surface-to-volume ratio [88]. However, anions like hydroxide and superoxide remained on the cell wall of bacteria disrupt comprehensiveness, which might ultimately destruction of the cell wall, ensuring the release of intracellular substances eventually leading to cell death. While a compound like H_2_O_2_ cause harmfulness to respiratory enzymes of the cells. The roughness of the nanoparticle's surface caused damage to the cell wall, resulting in increased plasma membrane penetrability to Ag^+^ and ZnO^+^ leading to toxicity to bacteria [89]. As compared to other nanoparticles like titanium oxide-zinc NPs, Ag/ZnO nanoparticles are more effective antibacterial agents. As a final point, it could be resolved that on the surface of bacterial cell wall generation of ROS species causes cell wall rupturing as the negative charged cell wall absorbs positive charged silver and zinc ions causing alternation in electrodynamic interaction ultimately leads to death [90]. Ag/ZnO NPs use against microbes and organisms causing disease in plants not yet been given more consideration. Meanwhile, the world goes through the problem of malnourishment and deficiency of food where these harmful organisms employ a role in demolishing significant crops; that is why, this could be a foremost area of concern regarding revolutionary exploration on Ag/ZnO nanoparticles, hence rising the importance of these NPs in their applicability [91].

### 5.2. Antioxidant Use

Plant-derived Ag/ZnO alloy NPs are being acknowledged for carrying antioxidant use as compared to metallic NPs. DPPH free radicals indicate antioxidant action depending upon quantity at an optimal concentration of synthesized NPs compared to ascorbate (standardized antioxidant anion). Additionally, solution change of color is due to NPs observed. Researchers showed that the combination of silver with zinc oxide forming plant-based nanomaterials increases their antioxidant capacity and their antiproliferative behavior causes the elimination of free radicals [92]. Thus, compared with monometallic silver and zinc oxide nanoparticles, bimetallic Ag/ZnONPs synthesized by greener chemistry using fenugreek plant had the more antioxidant capability. Ag/ZnO NPs as an antioxidant agent could be additionally applicable against vital fights like liver problems and cancer [93].

### 5.3. Cytotoxic and Cancer Curative Agent

**Figure 7:**
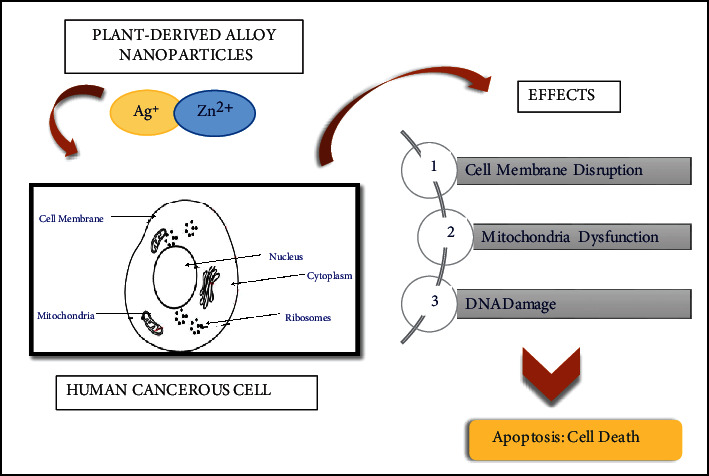
Adverse effect of NPs on cancerous cells leading cell death.

Plant-derived Ag/ZnO NPs had not been frequently used against human cancer cells, but these nanoparticles have the potential of acting against cancer when applied to various cancers of humans such as ovarian, liver, pancreatic, and lung cancer. Still, their efficiency is necessary to experiment. However, the function of silver/zinc-oxide NPs towards brain gliomas could be effective due to brain stem glioma, which acts as a barrier to drugs and makes cure utterly problematic. Biosynthesized bimetallic Ag/ZnO NPs from *Chonemorpha grandiflora* extract showed noteworthy outcomes for the investigation of toxicity. To evaluate the in vitro cytotoxic capabilities of silver/zinc-oxide alloy NPs different cell lines were utilized such as, MCF-7, HCT-116, and A-549. The phyto-synthesized silver/zinc-oxide alloy NPs exhibited different cytotoxic effects on these cell lines; however, toxicity to cell line depends on the dosage of nanoparticles. The practicability examinations also confirm that sustainability of cells significantly reduced with an increased amount of nanoparticles; moreover, cell sustainability that deceased in the following order was also recorded: MCF-7>HCT-116>A-549.  Figure 7 illustrates the adverse effects of NPS on cancer cells, also reported in the literature [94]. Silver/zinc oxide nanoparticles prepared by plants with specified nanosize and shape are more effective as anticancerous substances as compared to bulk size NPS. Reasonably, this is due to the availability of more quantity of plant extract that is reducing and stabilizing agents at the earliest steps of greener synthesis. Additionally, bimetallic nanoparticles showed a prohibitive influence on HepG-2 growth. As cancerous cells are dissimilar from regular cells, particularly concerning metabolic necessity, leading to diversified toxicity to cells [95]. Certain researches demonstrated that in cancerous cells, NPs having zinc-II cation produces reactive oxygen species which eventually destroys them [96, 97]. Death of cancer cells occurs due to zinc oxide-based nanoparticles as these NPs alter histone methylation and silver metal prompt programmed cell death in them by generating a maximum quantity of free radicals [98]. Because of more cellular absorption and withholding of plant-based silver-zinc oxide alloy nanoparticles, these NPs induce more toxic effects against HepG-2 cell line than normal cells like NIH-3T3 which was certainly time and quantity dependent. The nanometer size of particles showed the best toxic effect on human cervical cancer cells (HeLa), which can readily enter cells, generating reactive oxygen species resulting in cell mortality [99]. *Justicia adhatoda* derived silver/zinc oxide-based nanoparticles that positively charged silver, zinc metal ion produces more injuries as compared to negatively charged ion, because of ionic contact with cell membrane which is negatively charged. These metal ions result in cell membrane leakage and destruction of enzymes. Hence, silver/zinc oxide-based nanoparticles can be executed as the best operational and effective anticancer agents [100].

*Similarly*, *in vitro* cytotoxicity efficacy of phytosynthesized AZnO NPs was tested against human cancerous cell lines such as breast (MCF-7 and MDA-MB-231), colon (HCT-15) and lung (A549), and peripheral blood mononuclear cells (PBMCs). The highest cytotoxicity activity was observed against all the tested cell lines at 25 *μ*g/mL [101]. The cytotoxicity of various doses of biocapped Ag/ZnONCs (0.05, 0.1, and 0.2 mg/mL) to treat cell lines was evaluated by using cell viability test of human A549 cell line. The obtained results showed that concentrations of up to 0.1 mg/mL of biocapped Ag/ZnONCs induced no significant harmful to the cells [59]. Rad et al. demonstrated that low concentrations of Ag/ZnO NPs possess cytotoxicity and induce dose-dependent cell death [102].

Ag/ZnO NPs were examined for their cytotoxic effects in the cervical (HeLa) and ovarian (SKOV-3) carcinoma cell lines. The dose-dependent cytotoxicity was investigated by studying the cell viability assay, ROS generation, and adenosine triphosphate (ATP) contents when the cell lines were exposed to the ZnO/Ag composites for 48 h. Among the various concentrations ZnO/Ag composites produced, the 2.0 mg/mL ZnO/Ag 7.5 composite exhibited superior anticancer activity against HeLa and SKOV-3 cell lines. The cell viability and ATP contents were reduced significantly in a dose-dependent manner, whereas ROS generation was increased appreciably [103].

### 5.4. Antileishmanial Potential

In the tropical zone, leishmaniasis is a life-threatening disease and new techniques have been developed for its cure to gain popularity. Various biofabricated metal and their oxide NPs are being practiced [104]. Following the MTT assay, the heterostructure of plant-derived Ag/ZnO bimetallic nanoparticles showed the best activity against *Leishmania tropica* (KMH-23) as compared to monometallic ZnO nanoparticles [85]. Spherical shaped multimetallic NPS and Ag/ZnO synthesized by leaf extract of *Mirabilis jalapa* were also reported as having antileishmanial potential. Vector-based illness caused by the bite of mosquitoes such as dengue, malaria, and leishmaniasis bases the death of 0.6 million people worldwide. Regrettably, the research work explains the application of bimetallic Ag/ZnO nanomaterials as antileishmanial only whereas its use as antidengue and antimalarial agent is still required to be discovered [85].

### 5.5. Drug Delivery Application

Drug transfer to the specific sites is of significant use in biological and medical science aiming at delivering drugs to the targeted site, avoiding harmfulness to the normal cells present in their surroundings. By surface alternation of green synthesized nanoparticles, biological molecules like carbohydrates, protein, phenols, receptors, and drugs could be associated with plant-derived silver-zinc oxide NPs. This alternation discusses a particular role of bionanoassembles, making them capable of their application in medicine for specified drug delivery [69]. Hence, it is reasonable to specifically attack tumor cells by the mean of endocytosis, a lively targeting method [105–107]. Similarly, the outer surface of green processed nanomaterials is enclosed within biological compounds driven from plant extract, which could be transferred. Meanwhile, Ag/ZnO bimetallic nanoparticles are recognized for extraordinary proficiency of assemblage because of increased surface area, letting them bind with numerous chemical substances such as biological molecules and drugs. Thus, plant-based Ag/ZnO nanoparticles can take up or function by biomolecules of plant extracts, which can be exploited as a natural linker for drug delivery to the specified sites [108]. The biostable capability of biosynthesized silver-zinc oxide bimetallic nanoparticles headed to healthy and unhealthy cells building them more certain as a vector for delivering drugs. Green synthesized silver-zinc oxide-derived drug delivery system using the FDA-approved anticancer drugs could be effectively manufactured and applicable for enhanced therapeutic ability, compared with drug only. The better activity of the drug delivery system could be due to additional targeted effects, improved penetrability, and holding effects of these bimetallic nanoparticles. Analyzing biocompatibility of plant-derived Ag/ZnO nanoparticles, it is easy to certain the usefulness of biologically fabricated manufactured Ag/ZnO NPs as a competent vehicle for targeted drug delivery against cancer in the upcoming time [109].

### 5.6. Photocatalytic Use

**Figure 8:**
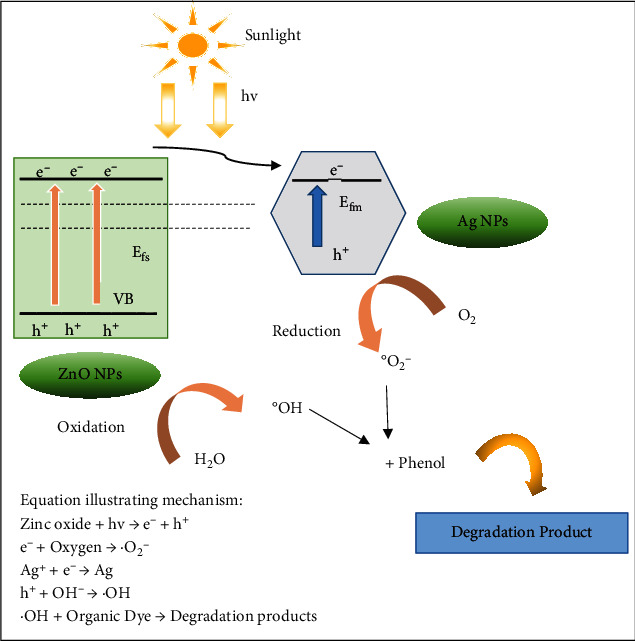
Demonstration of photocatalytic activity of Ag-ZnO nanoparticles.

Plant-based silver-zinc oxide bimetallic nanoparticles are popular for photocatalysis mainly for their reduction capabilities. The better mechanism of photocatalytic efficiency of green synthesized Ag/ZnO NPs can be described in Figure 8, as reviewed from research articles. Zinc oxide NPs grip energy photons that are equal or greater to holes, electrons, and bandgap energy produced in the valence band and conduction band. Meanwhile, the Fermi level of silver-zinc oxide is less than the energy level of zinc oxide conduction band; transfer of electrons could be done to silver from zinc oxide NPs. Henceforth, silver NPs could inhibit their recombination by trapping light-induced electrons. The electrons induced by light could produce ^∙^O_2_; however, holes of zinc oxide valence band could counter water resultantly producing a hydroxyl group. Both of these are the reason for the degradation of organic dye [110–113]. Hence, better photocatalytic efficiency of silver-zinc oxide nanoparticles could be recognized due to the establishment of Schottky junction at the interface of silver-zinc oxide, leading to upgradation of separation of charge carriers and resultantly minimizing recombination level, whereas these nanoparticles exhibited 90% photocatalysis with degradation of methyl orange dye after 2.5 hours under ultraviolet radiation, when discoloration was noticeable. Henceforth, nanosized materials with increased surface area and more transparency of silver-zinc oxide nanostructures might showcase a significant role in the improvement of photocatalysis [85, 114, 115].

### 5.7. Detection of Heavy Metals and Biosensor

In water, the existence of heavy metals like cadmium, lead, and mercury is unquestionably a major issue for decades. Lately, silver-zinc oxide NPs synthesized by biological method ensure its significant use for removal of inorganic pollutants like chromium (VI) [116]. The viability of this agent is experimentally checked in the ecosystem by the use of water samples. Additionally, these NPs could be used for the detection of other inorganic pollutants like lead and mercury in an ecosystem. Ag-ZnO nanomaterial-based sensors made by physical and chemical means are already in use for the detection and degradation of hazardous gases like NO_2_ from the environment [117]. Biosensors made of silver-zinc oxide bimetallic nanoparticles are being in use for the detection of uric acid in serum. The indicated usage could be further advanced for the detection of contaminations like inorganic pollutants, urea in water, and milk, respectively, mainly in developing and underdeveloped countries whereby common people use various harmful chemicals as agents for the thickening of milk [118].

### 5.8. Molecular Recognition Use

Biological molecules for example nucleic acid and proteins are well recognized for their application in coating silver-zinc oxide nanoparticles, illustrating conjugation of bimetallic nanoparticles with nucleic acid (Ag/ZnO-NA) like DNA and RNA. Meanwhile, this genetic material can bind with complementary strands; the NPS-NA can be used in molecular recognition of NA from solution [119]. Moreover, the capability of NA's self-assemblage enhances their recognition efficiency starting with sequence to specified molecular sites like proteins, cells, organs, and organisms. The identified application of bimetallic silver-zinc oxide nanoparticles could be extensively used for the detection of multiple deoxyribonucleic acid sequences to detect mutations in the polynucleotide sequence [120, 121].

### 5.9. Other Applications

Silver/zinc oxide-based bimetallic nanoparticles synthesized by the biological method are reported for several other usages, for example, sensor selectivity [122], photoluminance [123], excellent osteointegration, infection prevention [124], and anti-inflammation [125]. Ag/ZnO NPs prepared by propolis extract are reported for wound healing treatment [126], whereas Ag/ZnO NPs prepared by Prunus cerasifera are being used for pollutant degradation and in vitro biocidal performance [127]. The applications of silver-zinc oxide bimetallic nanoparticles are summarized in Table 1.

## 6. Lethality of Plant-Based Ag/ZnO Nanoparticles

The efficient performance of bimetallic Ag/ZnO NPs synthesized by plants depends on their various properties. We explained the diverse features and effective lethality by methods mediated by plant-derived silver-zinc oxide nanoparticles. Furthermore, production through ion leakage and generation of reactive oxygen species from the surface of Ag-ZnO nanoparticles also take place by their activation with light [128]. The production of reactive oxygen species from nanoparticles via excitation with light (with greater energy level or equivalent to bandgap energy), illuminated on the outer surface of nanoparticles, assisting elections of valence band towards conduction band while developing a hole in valence band. This makes pairs of electron-hole, responsible for (a) transfer of pairs to the surface of NPs, (b) permitting the incidence of oxidation-reduction processes of adsorbates, and (c) occurrence of oxidation taking place when oxidation-reduction efficiency of valence band is more positive as compared to redox efficiency of adsorbates. Likewise, electrons of conduction bands reduce adsorb types when oxidation-reduction efficiency is negative as compared to efficiency adsorbates. Certainly, the simple recombination is the feasible result of the creation of electron-hole pairs; besides the successful discharge of thermal energy (that can be additionally used for photothermal therapy), (d) at the time when oxidation-reduction processes are stimulated, recombination processes also take place responsible for the reduction of photocatalysis significantly. These circumstances hence lead towards maximum production of reactive oxygen species which in turn enhances the lethality of Ag/ZnO nanoparticles synthesized by biological means; therefore, they become more harmful [129]. Oxidative stress could be induced due to the generation of reactive oxygen species of maximum level, causing failure of cells in continuing usual physiological roles regulated by oxidation-reduction processes [130]. The destruction of functions and growth of cells is comprised of oxidative alternation of biological molecules like nucleic acid and proteins which generates protein radicals, breakage of DNA double-helical strands, lipid peroxidation, variations of gene expression by the initiation of transcription factors that are sensitive to oxidation-reduction processes, variation of inflammatory reactions by signal transduction, cell membrane fluidity, more permeability of charged elements, resulting destruction of the plasma membrane [131], effects to genetic material which added towards apoptosis and ultimately leads to death of cells [132]. The adverse effects of reactive oxygen species generated by biologically synthesized Ag/ZnO nanoparticles could be reduced. Secondary metabolites like polyphenols are well known as scavengers of reactive oxygen species and their presence as a coating on bimetallic silver-zinc oxide nanoparticles could alleviate the production of reactive oxygen species, or else this could have hindered the functioning of cells and causes damage to DNA [133, 134]. However, such types of nanomaterials were reported as enhanced photothermally efficient. Moreover, the size and morphological size of biosynthesized Ag/ZnO nanoparticles also affect their lethality. Minute-sized silver-zinc oxide NPs can easily enter the plasma membrane and effortlessly penetrate subcellular organelles resulting in inhibition of cellular functions due to the production of reactive oxygen species and high temperature, while uptake of nanomaterials decreases with an increase in nanomaterial size [135]. Cancerous cells cured by minute, photothermally efficient Ag/ZnO nanoparticles manufactured by the green approach go through structural variations including rupturing of the plasma membrane, cytoplasmic fluid leakage, and ultimately cell death [136]. These types of materials are also responsible for the generation of reactive oxygen species, which moreover increases the harmfulness of these nanomaterials [137]. Sometimes, lethality of plant-based silver-zinc oxide NPs is negotiated due to their aggregation with body liquids like blood, serum, and others such as cytoplasmic fluid having amino acids, proteins, vitamins, trace metals, and electrolytes, etc. These several constituents could corporate with NPs, altering physiological and chemical properties like size, charge, surface chemistry, and aggregation state through electrostatic transmission [138]. The aggregated form of NPS could affect their capability of interaction with cells and/or entrance into cells, hence adding complications to the system. Numerous proteins of plasma are coated on NPS intensively, and the chemistry of the outer side of NPs in plasma or growth media is altered as compared to nanomaterials that were synthesized initially [139].

Along with diverse therapeutic and medical uses, there are various hazardous effects linked with Ag/ZnO nanoparticles and nanocomposites and we need a basic understanding of harmful effects to deal with them correctly [140]. Bimetallic nanoparticles furtively take entry to the ecosystem through the soil, atmosphere, and water by numerous activities of human beings. Conversely, the use of silver-zinc oxide nanoparticles for the treatment of the ecosystem intentionally injects engineered nanoparticles into the lithosphere or hydrosphere, resulting in increased apprehension from all shareholders [141]. The benefit of plant-based nanoparticles is their high rate of reaction which could be developed into probable lethal issues by bringing adversative and harmful effects at the cellular level, rare micron-sized counterparts [142]. Researchers also explain that nanoparticles could enter living things during eating, drinking, or breathing, translocating to certain organs or tissues within the body where the nanoparticles have the opportunity to put forth the reactivity having toxic effects [143]. Though various studies illustrate the toxic and lethal effects of nanoparticles on auto- and heterotrophs at the cellular level, the studies about harmful effects with plant-based silver-zinc oxide nanoparticles on organisms to date are yet incomplete [144]. The application of Ag/ZnO nanoparticles produced by the green method in various products leads their entry to the hydrosphere, becoming the reason for dissolved Ag and ZnO, hence inducing hazardous effects on aquatic organisms like algae, bacteria, and fish [145]. The breathing system shows an exclusive objective for the potential lethality because of the statement that being portal of entrance for inhaled nanomaterials; it also takes the whole cardiac output [146]. Even with the quiet progress and initial approval of nanobiotechnology and the wide use of nanoparticles in the medical and therapeutic fields, the capability of adversarial effects to health due to long-term exposure of certain concentrations in humans and the environment has not still been developed [147]. Moreover, it is expected that in the future there would be an increased impact of metal-based nanoparticles on the ecosystem. The organization and coating around the macrobiomolecules, like proteins depending upon nanosize, morphology, surface charge, free energy, and functionalized groups, are one of the toxicological and lethal capabilities of Ag/ZnO nanoparticles [148]. Because of this conjugation, silver and zinc oxide creates adverse outcomes by protein unfolding, thiol crosslinking, enzyme activity loss, and fibrillation. Thermodynamic properties of nanoparticles favor their dissolution in organisms, or suspension media show the discharge of lethal ions as an additional paradigm [149]. In seawater and hard water, nanoparticles are inclined to aggregate and are importantly affected by the natural materials or by the organic matter of a specified type. The form of their dispersion would change the lethality and toxicity of nanoparticles in living organisms of their surrounding environment [150].

## 7. Trends

Including the subject of this reviewed appraisal and the significance of nanoparticles in the therapeutic and medical field, it is appropriate to remark on the latest improvement on the application of these bimetallic nanoparticles as probes or sensors and could be applied to various fields, for example, biology, chemistry, physics, plant sciences, engineering, and human health [151]. In recent years, various scientists had already generated remarkable studies, focusing beneficiaries of using colorimetric and inflorescence properties of silver-zinc oxide nanoparticles, permitting their application as chemosensor, as soon as nanobased systems had noteworthy photochemical and photophysical specialties, leading to a diversification of uses [152–154]. Likewise, discovered linkage of amino acid to a coumarin for coating bimetallic silver-zinc oxide nanoparticles resulted in the formation of the sensor system of chemo-based in vivo or in vitro practices [155]. In this particular condition, silver-zinc oxide NPs were superior because of their use in targeted drug delivery and in controlling the activity of proteins, among other applications. For verification of chemosensors, the researchers carried out studies of those nanoparticles carefully by use of various specified procedures, for example, X-ray or infrared diffraction, elemental analysis, and MS-spectrometry [156]. Hence, in the count to the creation of chemo-based sensors, it was promising to get hold of minute size, stable silver-zinc oxide NPs, allowing the building of a more urbane chemosensor comprising coumarin-protein-Ag/ZnO NPs [157]. While the synthesis of Ag-zinc oxide NPs and description of their characters along with their detection in samples are combined in the literature, however, this review focused on their production along with their therapeutic potential of these NPs by the mean of plant extracts, the greener biological approach, analytical procedures followed for completion of these goals, the assessment of the consequences like harmfulness of these nanoparticles in tissues of living organisms, permitting, for example, an original visualization of its delivery and movement in the cell and cell constituents [158]. Stressful reaction to NPS is the same area of interest, appearing as reasonably easy to develop, given a broad number of researches on the monometallic and bimetallic stress reaction in living organisms [159]. Further studies are conceivable which adds assessing the consequences of nanoparticles buildup in the tissues, organelle such as chloroplast and mitochondrial apparatus. Similarly, it is vital to recall that the quantity of NPs, tissue, duration of research, the form of an element, species, and chronic/acute type of treatment might outcome a dissimilar stressful reaction [160]. Experiments with other heavy and harmful metals are extensively performed, along with physiobiochemical and genetic attributes which have been examined [161]; however, when bimetallic nanoparticles are concerned, similar responses continue scarce. Furthermore, a new attribute about the effects of bimetallic NPs on the interaction of living organisms with physical surroundings is receiving devotion in upcoming studies, possibly giving importance to performing these experiments under natural conditions rather than controlled ones, which ensures valuable and favorable effects [162]. The intensive achievement of examiners conducting main experimental plans should include a multidisciplinary methodology. For example, this review explains a diversified number of studies; nonetheless, one feature that possibly seems to be deserted was the study of genetic changeability [163]. As different varieties, hybrids, and mutants of the same species might show different reactions to the same concentration of bimetallic nanoparticles, another feature that cannot be abandoned is the uptake of metals and their buildup in micro- and macroorganisms like autotrophs, which are utilized for human and animal intake [164]. This feature includes more concern due to the likelihoods of silver, zinc oxide metals, and bimetallic nanoparticles, inflowing into the food chain having the ultimate consequences on the health of human beings [165]. This sort of investigation would be crucial and supportive, and we need researchers dealing with nanoparticles for consideration of this feature leading to detailed studies related to uptake kinetics, buildup, and translocation of nanoparticles within the system directed, giving new vision and understandings about the application of bimetallic nanoparticles along with their consequences and harmfulness on organisms [166]. Straight linkage of this article for researches of upcoming time is necessary as well to remember that various species are taken as hyperaccumulation of silver and zinc oxide metal; their investigation and application in phytoremediation have been discovered, and a necessary amount of writings are accessible in the literature [167]. Though the similarity is not accurate about hyperaccumulation of metallic nanoparticles in living organisms, silver-zinc oxide nanoparticles would be the main component for documentation of mechanisms responsible for tolerance in stressful conditions and harmfulness of bimetallic nanoparticles [168].

## 8. Conclusion and Future Perspective

While the synthesis of Ag-zinc oxide NPs and description of their characters along with their detection in samples are combined in the literature, however, this review focused on their production along with their therapeutic potential of these NPs by the mean of plant extracts, the greener biological approach, analytical procedures followed for completion of these goals, the assessment of the consequences like harmfulness of these nanoparticles in tissues of living organisms, permitting, for example, an original visualization of its delivery and movement in the cell and cell constituents. With the rising confirmation of the significance of plant for synthesizing Ag/ZnO alloy NPs, research has been conducted with extract of parts like roots, rhizome, shoots, stem, bark, leaves, flowers, and seeds of various plants and is utilized for biomolecules like steroids, flavonoids, saponins, alkaloids, and secondary metabolites, having capacity of precursor salt reduction for the production of silver-ZnO nanoparticles. Secondary metabolites like polyphenols are well known as scavengers of reactive oxygen species, and their presence as a coating on bimetallic silver-zinc oxide nanoparticles could alleviate the production of reactive oxygen species, or else, this could have hindered the functioning of cells and caused damage to DNA. Bimetallic Ag/ZnO nanoparticles are of more importance, owing to industrial, medical, and environmental uses. Thus, bimetallic Ag/ZnO NPs synthesized by greener chemistry using fenugreek plant had more antioxidant capability.

Considering the vast significance of Ag/ZnO nanoparticles from the last decade and biological mean of synthesis for well-being and compatibility with living tissues, it is expected that silver-zinc oxide nanoparticles manufactured by the green method will be eventually useful in fields where these nanoparticles are prepared through other methods are already in use with high influence. Due to their effective microbial activity and biocompatibility at the same time, it can be predicted that Ag/ZnO NPs derived from plants would be more fruitful against microorganisms. By this approach, these nanoparticles could lead towards the development of a novel production of wide-scale antimicrobial drugs. The marketplace centered on Ag/ZnO NPs has now developed into a massive economy. Plant-derived NPs could offer a noteworthy part at this point. Because of more demand from application point of caution experiments, enormous scale researches, and development of vaccination and home-derived uses, plant-derived synthesis can propose a worthwhile and monetary outlet of these NPs. Hence, it is undeniably required that broad research experiments should be concentrated on manufacturing and modifying the plant-derived Ag/ZnO NPs to cope with the potentials usually put by chemically synthesized Ag/ZnO NPs.

## Figures and Tables

**Table 1:** Various applications of Ag-ZnO bimetallic nanoparticles.

Nanocomposite	Applications	References
Ag-ZnO	Antioxidant	[93, 94]
	Antimicrobial action	[83]
	Antibacterial property (destroy both gram negative and positive pathogenic bacteria)	[112, 169, 170]
	Bacterial detection and sterilization	[171]
	Antiseptics	[27, 127]
	Anticancer (destroy UVB induced skin cancer cell)	[95]
	Antileishmanial	[46]
	Anti-inflammatory	[125]
	Biosensors	[119]
	Drug delivery	[106]
	Heavy metal detection	[39]
	Molecular recognition, osteointegration	[42, 172]
	Photo catalysis	[114]
	Pollutant degradation	[128]

## Abbreviations

Ag: Silver
ZnO: Zinc oxide
Ag/ZnO NPs: Silver/zinc oxide nanoparticles
UV: Ultraviolet
MRI: Magnetic Resonance Imaging
Nm: Nanometer
*M. luteus*: *Micrococcus luteus*
*E. coli*: *Escherichia coli*
*S. aureus*: *Staphylococcus aureus*
*P. aeruginosa*: *Pseudomonas aeruginosa*
*S. epidermis*: *Staphylococcus epidermidis*
*B. subtilis*: *Bacillus subtilis*
*K. pneumonia*: *Klebsiella pneumonia*
ROS: Reactive oxygen species
HCT-116: Human Colorectal Carcinoma
A-549: Adenocarcinomic human alveolar cells
HepG-2: Human Hepatoma Growth
MTT assay: 3-(4,5-Dimethylthiazol-2-yl)-2,5-diphenyltetrazolium bromide assay
FDA: Food and Drug Administration
NPS-NA: Nanoparticles conjugated with Nucleic Acid
MS-spectrometry: Mass spectrometry.

## Supplementary Materials

Supplementary Materials The supplementary file is a graphical abstract/summary of the article which represents the role of medicinal plants and various approaches for synthesis of bimetallic particles. Furthermore, the figure also shows the different applications of the phytosynthesized bimetallic nanoparticles.
