# Supplementary material for: Plant-Based Bimetallic Silver-Zinc Oxide Nanoparticles: A Comprehensive Perspective of Synthesis, Biomedical Applications, and Future Trends
Source: Biomed Res Int. 2022 Apr 30;2022:1215183. doi: 10.1155/2022/1215183 (PMC9078794; doi:10.1155/2022/1215183)
Supplement: Supplementary file 1 [file 1215183.f1.docx]

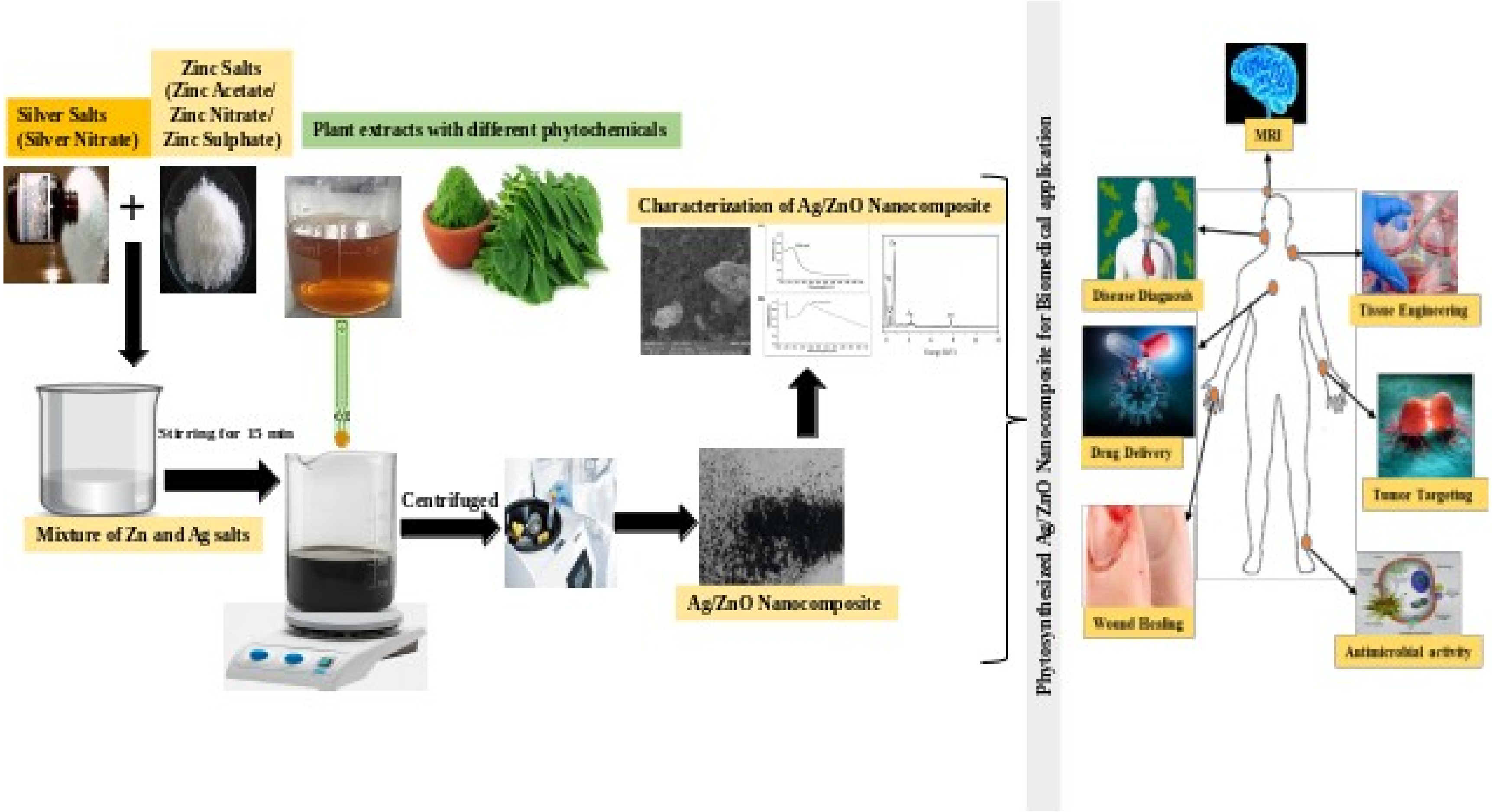
Silver Salts (Silver Nitrate)
Zinc Salts (Zinc Acetate/ Zinc Nitrate/ Zinc Sulphate)
Plant extracts with different phytochemicals
Characterization of Ag/ZnO Nanocomposite
Stirring for 15 min
Mixture of Zn and Ag salts
Centrifuged
Ag/ZnO Nanocomposite
Phytosynthesized Ag/ZnO Nanocomposite for Biomedical application
MRI
Disease Diagnosis
Tissue Engineering
Drug Delivery
Tumor Targeting
Wound Healing
Antimicrobial activity
